# Design, development, and local production of lipid-based nutritional supplements to enhance the complementary feeding diet: A model for collaboration for a feeding trial in Bangladesh

**DOI:** 10.12688/gatesopenres.13673.1

**Published:** 2022-09-21

**Authors:** Rebecca Merrill, Saskia de Pee, Tahmeed Ahmed, Klaus Kramer, Nahid Hossain, Nuzhat Choudhury, Britta Schumacher, Georg Steiger, Shahid Minhas, Abu Ahmed Shamim, Saijuddin Shaikh, Rachel Fuli, Parul Christian

**Affiliations:** 1 Center for Human Nutrition, Department of International Health, Johns Hopkins Bloomberg School of Public Health, Baltimore, Maryland, 21205, USA; 2 World Food Programme, Rome, Italy; 3 icddr,b, Dhaka, Bangladesh; 4 Sight and Life Foundation, Basel, Switzerland; 5 DSM Nutritional Products, Kaiseraugst, Switzerland; 6 Centre for Non-communicable Diseases and Nutrition, BRAC James P Grant School of Public Health, BRAC University, Dhaka, Bangladesh; 7 JiVitA Project, Gaibandha, Bangladesh

**Keywords:** micronutrient, lipid-based nutrient supplements, maternal and child, malnutrition, multiagency collaboration

## Abstract

**Background**: Lipid-based nutrient supplements (LNS) are effective for treating childhood wasting and for preventing stunting, wasting, and anemia, but large-scale production and programmatic use are a barrier. Locally-developed and produced LNS may be more affordable and reduce logistical procurement and importation hurdles, while promoting private sector engagement and partnership.

**Methods**: In northwestern Bangladesh, we conducted a community-based trial of complementary food supplementation to test its efficacy to reduce childhood stunting. Two locally-developed, small-quantity LNS (20g/day, rice-lentil and chick-pea based) were designed, developed first at small scale in the ‘kitchen’ laboratory under controlled conditions, followed by taking them to a local food manufacturer for larger production for the study. We describe here the partnership, required expertise and capacity, experiences, and lessons learned that made this uniquely complex undertaking possible

**Results**: Key steps in the journey included addressing the dynamics of clear communication between partners, executing on carefully assigned tasks and roles, correcting course when needed, and maintaining timeliness and roadmaps. Knowledge of food science and technology was key in solving many food-production challenges that were encountered in taking the laboratory recipe to the factory. Factory production was established and had to meet quality and hygiene criteria set for young children.

**Conclusions**: We provide documentation of this experience as a model to describe the various steps and considerations and what is entailed in local LNS production. We highlight the importance of a well-conceived collaboration with clear roles that created a ‘win-win’ situation for all partners for achieving common goals, establishing improved technology at the factory, and building new capacity to produce such products for children in a low resource setting.

Key words: micronutrient, lipid-based nutrient supplements, maternal and child, malnutrition, multiagency collaboration

## Introduction

The global burden of childhood stunting at 149 million worldwide is associated with a myriad of risk factors, including inadequate quality of complementary foods starting at 6 months of age and may increase the likelihood of severe outcomes including mortality ^1, 2^. Globally, in addition to counseling for infant and young child feeding practices, provision of nutritious foods for young children during the complementary feeding period from 6 months of age through the end of breastfeeding has long been part of nutrition and food aid programs ^3, 4^. Currently, among the complementary foods (CFs) that exist, the United Nations World Food Programme (WFP) distributes them in the form of enhanced fortified blended foods such as SuperCereal Plus which requires cooking or other ready-to-eat lipid-based nutrient supplements (LNS) in settings where children face a high risk of chronic and acute malnutrition due to natural disasters, conflict, or seasonal or chronic hunger ^5^.

Interest in special nutritious foods specifically designed for complementary feeding has largely emerged with the development of special high-energy and nutrient-dense therapeutic foods for the local management of severe acute malnutrition. The emergence of those novel products and the gradual shift in global food assistance toward settings of protracted disasters have also sparked interest in refining traditional food aid products to better meet the needs of the complementary feeding period ^5, 6^. Lipid-based nutrient supplements (LNS) are CFs designed to prevent wasting, micronutrient deficiencies, and stunting through regular consumption of small quantities provided in 20 to 50 g servings ^7^. In a recent Cochrane review of 17 randomized controlled trials (RCTs) that included 23,200 children and a meta-analysis of individual participant data from 14 studies with more than 37,000 children, supplementation with fortified small-quantity (SQ; providing 120 kcals and 2.6 g of protein) LNS as complementary food supplements showed significant reduction in child stunting, wasting and anemia ^8, 9^ as well resulted in a 27% reduction in child mortality ^2^. In the recent Lancet nutrition series, these evidence-based interventions are recognized for impacting the global burden of undernutrition ^10^. Lipid-based nutrient supplements also support improved linear growth better than fortified food blends in some settings and with some formulations ^11– 15^. Internationally distributed SQ-LNS products such as Nutributter and LNS-Medium Quantity (MQ; providing 275 kcals and 5 to 8 g of protein) such as Plumpy’doz (Nutriset, France) are ready-to-eat peanut-based formulations that are available for use as complementary food supplements but may likely be more expensive than locally manufactured products, although not many settings have local production capacity of such products. Additionally, local wariness of imported nutritional products and of population habituation to complementary foods that are not locally available, and low acceptability of peanut-based LNS are challenges to sustaining wide distribution of globally-available LNS products ^16^. Further, even with successful local production to date, using internationally developed recipes, some populations may not prefer the products because in some settings, such as Cambodia and Vietnam, peanut-based LNS was not well accepted ^17, 18^. To address this, some local programs, including Pakistan’s program, use more preferred local chickpea-based LNS formulations, such as Wawamum ^4^. In Bangladesh, social enterprises have recently started commercial production of economical LNS such as Hashi Khushi for children ( Frontier Nutrition).

Interest is mounting in developing and distributing alternative, locally or regionally developed and produced LNS through governmental, non-governmental, or commercial channels independent of food assistance programs, especially where undernutrition is highly prevalent. In support of this goal, designing more cost-efficient and efficacious solutions to enhance the quality and accessibility of these products in low-resource settings is paramount. Further, there is a need to develop LNS tailored to the special demands of the complementary feeding period ^19^. From 2012–2014 we conducted a community-based RCT, implemented at the JiVitA Project site in rural northwestern Bangladesh, involving over 5300 children who received different types of complementary food supplements, including two locally designed and produced rice-lentil and chickpea based LNS along with one fortified blended food, and one ready-to-eat LNS ^20^. The study evaluated the impact of supplementation on improving growth and reducing stunting, wasting, and improving micronutrient status if consumed daily by children from 6 to 18 months of life.

**Figure 1.**
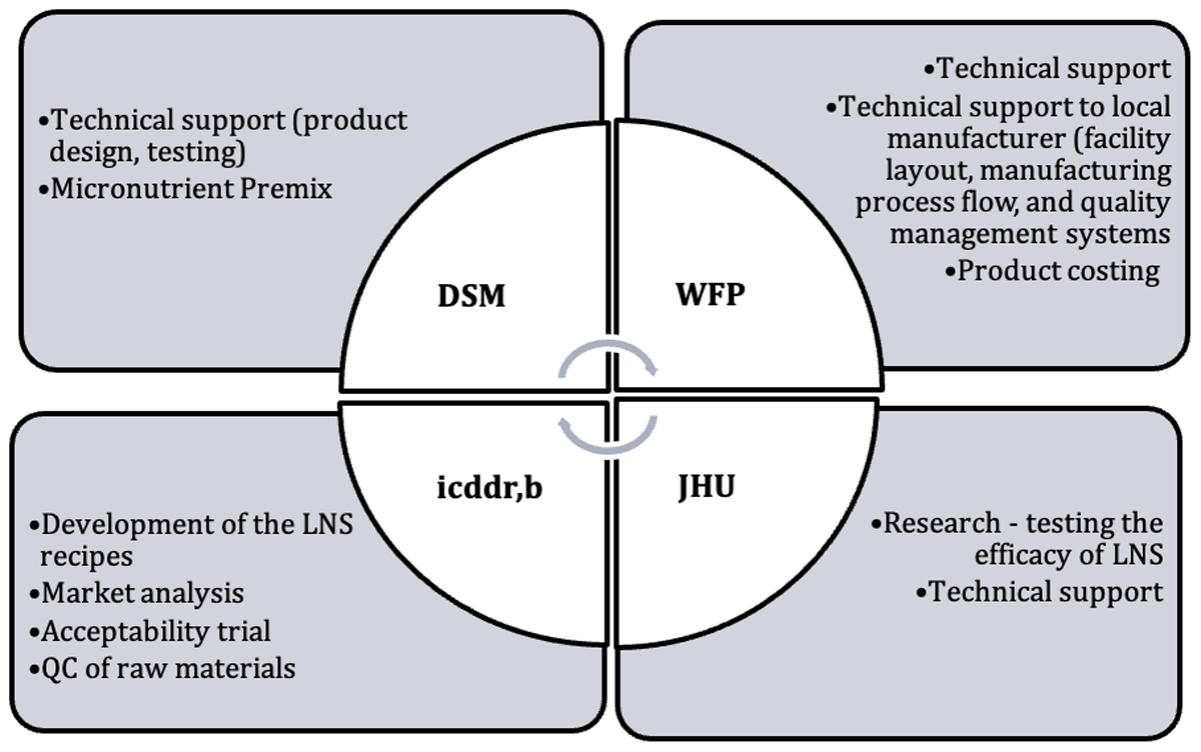
Participating organizations and general responsibilities.

The novel aspect of this study entailed design, development, and production of 2 local LNS products, prior to testing them in the RCT. In order to accomplish this work, 1.5 years prior to the RCT, a group of organizations developed a strategic partnership ( Figure 1). The group of organizations and agencies included an academic institution, the Center for Human Nutrition of Johns Hopkins University; a community-based research project, JiVitA, of a national non-governmental organization, Johns Hopkins University, Bangladesh; an in-country, international research institution with expertise in nutrition and food science, the International Center for Diarrhoeal Disease Research, Bangladesh, icddr,b; a UN agency involved in international food assistance, World Food Programme (WFP); the manufacturer, Olympic Industries; a nutrition ingredient company, DSM; and the nutrition think-tank, technical partner Sight and Life Foundation, Switzerland. Each partner provided unique expertise and strength critical to the overall objective to develop locally-produced and -sourced LNS designed to address the nutritional needs of the country’s children. In addition to the technical partners, in-kind support in the form of micronutrient premix for the food supplements was provided by DSM, Basel, Switzerland, and Plumpy’doz was provided by Nutriset (Maulany, France).

This paper presents the journey of the development and production of two local LNS products through partnership with a local food manufacturing company, key challenges faced and overcome, lessons learnt, and the central role the collaboration played in project success. This partnership design and experience serves as a model for achieving a similar goal in another setting.

## Methods for collaboration experience

We describe the experience and work that led to the successful design, production, and supplementation of children with two LNS produced using local ingredients and manufacturer. Information for this paper was gathered from experiences documented in meeting minutes, study protocols and standard operating procedure manuals, regular reports and communication between the partners, and site visits to the production plant. Additionally, at the completion of the field trial, all colleagues were interviewed by soliciting written responses through a standard survey for their opinions and experiences. The study received ethical approval from the Ethical Review Committee (ERC) at icddr,b, Bangladesh and the Institutional Review Board (IRB) of the Johns Hopkins Bloomberg School of Public Health, MD, USA on August 3, 2011 (reference no. 00003703).

### Cultivating collaboration and establishing a communication plan

In order to develop, produce, and test a locally-produced LNS, a range of expertise and routine and transparent communication among the partners with established roles and agreed upon responsibilities was required. The group recognized that convening multiple partners from numerous locations on a regular basis was a critical step towards establishing effective communication and a forum to resolve issues, both of which were invaluable for supporting the overall project objectives. Additionally, the partners felt that developing a co-owned collaborative framework was important for launching and maintaining the effort. 

**Figure 2.**
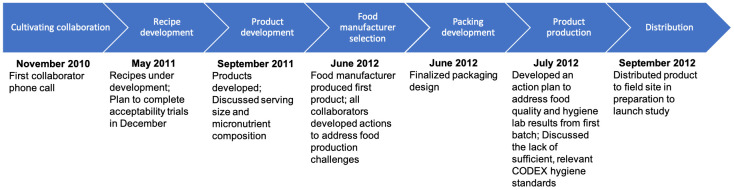
Timeline of project milestones.

 During the first partners’ conference call, occurring 13 months before the expected trial launch date, participants clarified roles and communication strategies and established terms of reference to reflect agreed-upon achievable deadlines in light of project funding and in-kind support, ongoing, non-project related activities, and anticipated challenges ( Figure 1, Figure 2). During subsequent meetings, scheduled monthly on average, partners revisited strategies and deadlines, presenting openly their challenges, namely those that would impact achieving the objective of producing the LNS products at the industrial level within the set goal of 1 year. Establishing early in the collaboration a cadence of meetings and anticipated outputs and deliverables ensured open vetting as a group of each partner’s accomplishments, current and projected bottlenecks, strategies to address bottlenecks, and, when necessary, consequent adjustments to the overall timeline.

In addition to the communication among partners, it was critical to ensure the Bangladesh government was involved and informed. Thus, partners prioritized presenting the activity to and maintaining routine communication with key public health and nutrition stakeholders and government agencies. Toward this objective, an initial partner task was established focusing on building country-based knowledge about and support for the complementary food supplementation project within the Ministry of Health and Family Welfare (MoHFW), the Bangladesh Institute of Public Health Nutrition (IPHN), and the Bangladesh offices of Alive and Thrive, Nutrition International, UNICEF, and the US Agency for International Development (USAID), among other identified key stakeholders. Additionally, as one of the first actions, the partners introduced the project objectives to the existing Nutrition Working Group, comprised of representatives of the key nutrition partners including UN Agencies, development organizations, civil society, and public sector institutes. This dialogue led to cultivating a venue for answering questions and seeking inputs on the project. A Technical Committee was also formed and led by a leading scientist (TA) and member of a partnering organization, icddr,b, and was composed of child nutrition and health leaders and scientists in the MoHFW, and in academic, medical, and public health fields. This Technical Committee informed and sought guidance from our collaborative project. 

### Designing a region- and population-specific LNS

***Recipe development.*** An important aspect of developing an LNS as a complementary food supplement for rural Bangladeshi children 6–23 mo of age, guided by existing international products, required creating a recipe that incorporated locally-available, culturally recognized and acceptable ingredients. The partners needed to ensure the products were fortified with multiple micronutrients at agreed upon levels sufficient to bridge the requirement gap in this age group while not adversely affecting the organoleptic or shelf-life qualities of the final product. Detailed consultations among nutritionists and food scientists were needed to finalize the target composition of the LNS including the amount of animal source (dairy) protein in the supplement recipe as well as levels of nutrients included in the premix. Further, the partnership leveraged their broader networks of experts, including a food technologist with WFP based in Pakistan (SM) who was supporting the production and distribution of a chickpea-containing LNS. The food technologist participated in developing the Bangladesh LNS version and provided other technical assistance throughout the LNS production activities.

***Product development.*** Collaborators affiliated with the icddr,b food processing laboratory played a seminal role in the development of the LNS products. Details about how these products were designed, developed, and finalized are described in detail elsewhere ^21^. In brief, the icddr,b food technologists created two recipes – one with a rice and lentil base and another with a chickpea base. The formulations were designed as LNS and thus in addition to the grains, the products contained vegetable oil, and dried skim milk powder was the source of animal protein in addition to the plant proteins found in the grain and cereal grains. Producers could procure the ingredients in bulk locally, at a reasonable price, and at a standard required to maintain a consistently high quality LNS. 

Once the new formulations were defined and the nutrient composition of the final products measured, the partners determined the desired micronutrient composition of the LNS which would be met by adding a premix to the formulation. The food technologists on the team participated actively in the composition discussions to provide guidance on ensuring the levels and ratios of certain vitamin and minerals and their influence on product stability and organoleptic properties, while the nutritionists ensured fulfilling daily requirement gaps based on a diet of breastmilk and complementary foods. To specify the micronutrient composition, the following aspects were considered: desired portion sizes of the LNS and daily recommended micronutrient intake consumed through one serving by children; the ratio of minerals (such as iron and zinc) to preserve bioavailability; levels of nutrients that could influence organoleptic properties of the product; shelf-life of the product; accounting for micronutrient amounts intrinsic in the LNS ingredients, etc. Once finalized, DSM supported the production and provision of the appropriate micronutrient premix. The chemical form of the micronutrients was chosen considering their application in the specific food matrix, including bioavailability, stability, and organoleptic properties.

The consensus among the partners was that a daily portion of the LNS should provide energy levels found in SQ-LNS (125 kcals) to fill the energy gap in home-based traditional diet. However, because this food would be distributed to a population consuming a diet low in micronutrient content and variety, they devised the premix to provide 75 to 100%, on average, of vitamin and mineral requirements. For a year-long supplementation starting at 6 mo of age, provided by the JiVitA-based RCT, the partners agreed that the LNS would provide children 6–11 mo of age 125 kcals per day through a single sachet of SQ-LNS and children 12–17 mo with 250 kcals per day through two sachets.

Once the food processing lab at icddr,b finalized the recipes, they shared samples with the partners. We then proceeded to conduct an acceptability trial of the two products among the target group of young children in the rural Gaibandha district where the RCT was to be conducted. The results of this study showed high level of acceptability for both products ^21^. Concurrent with the acceptability trial, the icddr,b team executed a shelf-life study to determine, among other characteristics, safety (microbes, toxins, heavy metals), water activity, and oil separation tendencies of the products.

### Developing a production and distribution plan

***Selecting a food manufacturer.*** Partners had to select a local food manufacturing company interested in and capable of producing the foods in the quantities required for the RCT. This production had to adhere to strict, established food product hygiene standards and requirements ^22^ for children under 2 years of age.

The other success factor for this phase of the project was creating clearly defined tasks and responsibilities for each partner in the manufacturing process. More specifically, a detailed LNS production plan map with a timeline of tasks and individual or groups named responsible for completing tasks guided the effort. Clear reporting from each individual in regular meetings resulted in establishing a good understanding of whether the plan was being executed as anticipated and for course corrections decided collectively.

WFP, a global expert in procurement, logistics, transportation, and storage, played an important role in selecting a suitable and interested manufacturer, leveraging existing collaborations for their fortified biscuit programs. The partners formed a Task Force from the collaborating organizations who developed a shortlist of potential food manufacturers based on their available product range as well as having access to the required equipment, facilities, and quality assurance methods for LNS production.

The selected food manufacturer would have to assume a variety of responsibilities for the study, which required production of a total of 12–15 metric tons each of the two products including creating a separate production line that met the factory specifications for products designed for children less than 2 years old and take this on without knowing if there would be future demand for the product. In addition, before initiating any production, the factory would need to undertake product development efforts to adapt the recipes from the kitchen-based to the manufacturing versions.

The partners approached the five listed companies to request meetings with senior teams and visits to production units to assess the companies’ capacity and willingness to produce food products for young children. Notably, none of the companies, nor any in Bangladesh, were engaged in producing food for children under 2 years of age. Two of the three companies were not interested in initiating the activity due to the resources required to achieve and maintain the strict, high standards, especially without promise of future business and associated revenue for such products. Based on the Task Force assessment, Olympic Industries, Dhaka, one of the largest manufacturers and distributors of biscuits in Bangladesh, met the various criteria including an expressed interest to participate. Olympic was also the most open to the collaboration and was willing to make modifications to their production equipment and lines to meet the needs for production of the complementary foods for children.

The partners established a memorandum of agreement with Olympic Industries with agreed upon payment of the contractual services, largely coordinated by WFP including early investments to set up a separate production room, dedicating equipment, purchase of clean raw ingredients, among others. Following this, the company initiated setting up a production line assembly, modifying existing machines and their location, sourcing raw consumable and package materials, and developing a hygiene and quality assurance plan. The partners supported and monitored these activities. To provide more specialized support, an independent food technologist from Thailand who was an expert in food microbiology and large-scale manufacturing processes especially related to quality control, texture, appearance, and viscosity analyses visited the production site every other week, accompanied by icddr,b and WFP-Bangladesh colleagues, to provide technical inputs during the early phases of the factory production. A main highlight of the project was the successful technology and knowledge transfer from a food processing lab to a local processed food producer for the production of foods for young children requiring markedly high standards. However, one of the challenges faced was that the time required to establish the specialized factory lines was longer than expected.

Olympic Industries first undertook a trial production run, which facilitated addressing numerous issues including better defining the roasting duration of the lentils and chickpeas, adjusting the overall moisture content and size of ground particles, adjusting fluidity to change the products’ stickiness, shifting the recovery rate, and improving extrusion. Simultaneously the partners and company leadership refined hygiene protocols and completed microbiological testing on initial product during the trial.

***LNS packaging.*** Designing single serving sachet packaging for SQ-LNS (i.e. 25–28 g/sachet) required considering the type of material, product stability, labeling, cost, transportation, and storage. After much discussion, the partners finalized sachet packaging material as triple laminated with low-density polyethylene as a compromise between cost and ability to extend shelf life. As required by Codex Alimentarius, a set of food standards established by the World Health Organization (WHO) and the Food and Agriculture Organization of the United Nations (FAO), the partners included labeling of ingredients on each individual serving sachet. Additionally, they decided on the size of each sachet, the number of sachets per box (labelled with ingredients and storage instructions), and the number of boxes in a shipping box. Importantly, the partners ensured that the sachet marked an ‘easy tear’ location after hearing that other regional programs learned during implementation that community members resorted to using razors when unsure how to open the product, which could introduce contamination.

***Maintaining quality and hygiene standards.*** Adhering to Codex Alimentarius requirements was important for LNS designed for young children. Thus, the partners carefully scrutinized manufacturing standards on quality and hygiene and put quality controls in place. The various elements of this component included a) factory line cleaning and hygiene protocols, b) sourcing of affordable but high-quality local ingredients, c) batch-specific microbiological and contaminant testing and approval, and d) shelf-life testing and expiry date labeling.

Olympic Industries management instituted strict protocols for their staff to support proper hygiene. Between batch productions, factory staff rigorously and thoroughly cleaned the machinery to remove any contamination that could have built up. One challenge in Bangladesh was to procure ingredients of consistently high quality. Further, it was not possible to trace the purchased product back to a specific region or farm, as a vestige of the complex, multistep supply chain system in rural, agricultural Bangladesh. The small scale of production by family farms in Bangladesh meant that a bulk purchase of 300 kilograms of lentils was likely composed of product from more than 10, if not a multiple of 10, farms. This aspect of the supply chain highlights the challenges producers may face when trying to procure ingredients of consistent quality at scale. To address this challenge, Olympic Industries routinely procured from a wholesale market three hours away from Dhaka. During each market visit, the procurement lead identified the vendor with the highest quality product. Of note, milk powder and the vitamin and mineral premix were sourced outside of Bangladesh.

Related to the raw ingredients, one of the first challenges the partners faced was that the first few batches had higher than permissible levels of mold contamination. This resulted in increasing rigor in purchasing quality ingredients and in pre-picking, -sorting, and -sifting lentils and chickpeas. These consistent actions helped reduce the risk of contamination.

One of the most persistent challenges the partners faced was achieving and maintaining appropriate microbiological and contaminant standards in the final product. Due to not having local laboratories that could certify the hygiene quality of the products, Olympic Industries and the partners had to ship samples from each lot to the SGS Laboratory in France for testing and certification. At the time of this activity the icddr,b laboratory followed ISO 17025 but had not concluded global accreditation. This testing and certification process in France was time consuming requiring two or three days for sample shipment to SGS and at least 10 days for analyses and report generation and cost over $1000 per sample. Thus, the lack of availability of a credible local laboratory for contaminant testing increased the cost of and time required for testing and ultimately production at scale. This type of delay regularly challenged the supply pipeline and required a dedicated budget.

Another significant hurdle was not having Codex-specified cut-offs for microbiological levels at the time this project was undertaken. Fortunately, we were able to apply WFP’s established cut-offs. The food technologists discussed each report and determined acceptability of the lot. In the event a batch was classified as contaminated, Olympic burned the whole batch, often about three metric tons worth (this occurred for four batches in all) and started over again with production and SGS contaminant testing. Contamination was largely due to elevated total plate count, total coliforms, molds, and *Bacillus cereus* either unexplainable or possibly resulting from a number of factors ranging from a contaminated batch of ingredients to a protocol breach with staff hygiene practices.

## A model for replication

Recent evidence for the use of SQ-LNS for prevention of stunting, wasting, and anemia calls ^8^ for wider programmatic use of such products in high burden countries ^1^. At scale production and procurement of complementary food supplements will require country and regional manufacturing capacity to meet the demand of wide-scale use. While the study collaborators did not anticipate several challenges faced throughout the process of developing and producing two new, local LNS products for trial purposes, the lessons learned from our experience can help provide a model to replicate and improve production in other low-resource context including partnerships with food manufacturers as well as creating systems for testing the quality and safety of the products ( Table 1).

**Table 1.** Key challenges and recommendations.

Challenge	Recommendation
Ensuring appropriate and required skill-sets within the collaboration	Start initiatives early to select collaborators with expertise and seek out additional members quickly if a needed expertise is not represented
Maintaining efficient project management and timeline	Routinely discuss partner roles and responsibilities, establish task ownership and accountability, and organize regular meetings and routine communication.
Establishing a manufacturing partner in-country and a production line	Landscape potential partners both in terms of willingness to collaborate and capacity; consider a large-scale production and business case for the private partnership for both reasons of commitment and sustainability Communicate with the producer to develop a comprehensive framework for procurement, programming, monitoring, feedback and improvement for consistent supply
Transferring the ‘kitchen scale’ recipe to industrial scale	Consider industrial scale production early on, i.e. during recipe development and have expertise who helped develop the kitchen scale recipe present during the transition to industrial scale production
Testing batches routinely for contamination	Find a local or regional lab that adheres to global standards (ISO) to save time and perhaps cost.
Managing budget considerations as timeline extensions have financial implications	Establish a timeline early and routinely update the timeline based on current and projected progress while communicating changes with funders.

## Conclusion

We launched a partnership to conduct a RCT of different complementary food supplements including LNS that we challenged ourselves to manufacture locally. This added enormously to the complexity of the study. By building a unique collaboration across a range of expertise and adapting to contextual factors in Bangladesh, our partnership successfully designed, developed, and manufactured two local LNS products for testing for their efficacy in reducing child undernutrition and for future use in the country and region as the LNS showed showed significant reductions in stunting. In addition to producing 27 MT of a food tailored to young children within a two-year activity timeline, additional highlights included establishing a unique forum where partners could communicate, discuss challenges, and course-correct by leveraging a diverse set of skills required throughout the process from understanding food science, to child nutrition and development, to logistics and the supply chain, to food production. As stated by one of the partners, “we have jointly been able to solve all of the many challenges that developed during the preparation and implementation of food production. This success is because of the complementarity of the partners, the constructive approach, and strong commitment of all parties to the project.” Documenting this experience, which usually is not carefully done, will hopefully lead to its use for future such efforts in low-resource settings for impacting the global burden of childhood undernutrition.

## Data Availability

No data are associated with this article.
