# Supplementary material for: Design, development, and local production of lipid-based nutritional supplements to enhance the complementary feeding diet: A model for collaboration for a feeding trial in Bangladesh
Source: Gates Open Res. 2022 Sep 21;6:122. [Version 1] doi: 10.12688/gatesopenres.13673.1 (PMC9633643; doi:10.12688/gatesopenres.13673.1)
Supplement: Peer review history

# Peer review: Reviewer response for version 1

**DOI:** 10.21956/gatesopenres.14958.r32594

**Published:** 2022-11-03

**Authors:** Bindi Borg

**Affiliations:** 1 Macquarie University, Sydney, NSW, Australia

There is a paucity of protocols describing the development and testing of ready-to-use supplementary and therapeutic foods and even fewer papers describing the process of that development. Thus, this paper is an important contribution to a very small body of literature. The authors rightly note that the experience and challenges are not routinely documented. The paper describes in useful detail the challenges and lessons learnt in the process of developing and testing specialised nutritious foods in low-resource settings, which will be of invaluable assistance to others attempting similar projects. 

 It would also have been useful to compare and contrast the Bangladesh experience with those similar experiences elsewhere that have been documented, notably in Cambodia (Borg *et al.*, 2019 ^1^ and Sigh *et al.*, 2018 ^2^). Adding this element would be of great benefit to future researchers undertaking the task of developing and testing specialised nutritious foods in low-resource settings.

Does the article adequately reference differing views and opinions?

Partly

Are all factual statements correct, and are statements and arguments made adequately supported by citations?

Yes

Is the Open Letter written in accessible language?

Yes

Where applicable, are recommendations and next steps explained clearly for others to follow?

Yes

Is the rationale for the Open Letter provided in sufficient detail?

Yes

Reviewer Expertise:

Development of locally produced ready-to-use supplementary foods

I confirm that I have read this submission and believe that I have an appropriate level of expertise to confirm that it is of an acceptable scientific standard.

## References

[1] : Development and testing of locally-produced ready-to-use therapeutic and supplementary foods (RUTFs and RUSFs) in Cambodia: lessons learned. *BMC Public Health* .2019;19(1) : 10.1186/s12889-019-7445-2 10.1186/s12889-019-7445-2 31470824PMC6717373

[2] : Development and Acceptability of Locally Made Fish-Based, Ready-to-Use Products for the Prevention and Treatment of Malnutrition in Cambodia. *Food Nutr Bull* .39(3) : 10.1177/0379572118788266 420-434 10.1177/0379572118788266 30092653

# Peer review: Reviewer response for version 1

**DOI:** 10.21956/gatesopenres.14958.r32648

**Published:** 2022-10-18

**Authors:** Gerard Bryan Gonzales

**Affiliations:** 1 Nutrition, Metabolism and Genomics Group, Division of Human Nutrition and Health, Wageningen University and Research, Wageningen, The Netherlands

The manuscript describes the route the authors have taken to design, develop and trial a lipid-based nutrient supplement in Bangladesh. The letter details the various aspects of building partnerships with local and international institutions, think-tanks and companies in order to produce more accessible LNS. The manuscript is very well-written and is a great example and inspiration for researchers who aim to develop nutrition interventions in low- and middle-income countries.

 Although not a ground for revision/rejection, I would appreciate this letter even more if description of the warehousing and storage of the products are also described. How do we maintain the stability and shelf-life of the products during storage? Do we need large temperature-controlled warehouses? Nonetheless, I believe this paper is an important manuscript.

 As the letter only explains their experiences, there are no methodological, statistical nor technical aspects to be reviewed. I therefore recommend indexing this paper as it is.

Does the article adequately reference differing views and opinions?

No

Are all factual statements correct, and are statements and arguments made adequately supported by citations?

Yes

Is the Open Letter written in accessible language?

Yes

Where applicable, are recommendations and next steps explained clearly for others to follow?

Yes

Is the rationale for the Open Letter provided in sufficient detail?

Yes

Reviewer Expertise:

child malnutrition, food technology, molecular epidemiology, food chemistry

I confirm that I have read this submission and believe that I have an appropriate level of expertise to confirm that it is of an acceptable scientific standard.
